# Alkyl ferulic acid esters: Evaluating their structure and antibacterial properties

**DOI:** 10.3389/fmicb.2023.1135308

**Published:** 2023-02-10

**Authors:** Wei Song, Jiaying Xin, Chong Yu, Chungu Xia, Yu Pan

**Affiliations:** ^1^Key Laboratory for Food Science and Engineering, Harbin University of Commerce, Harbin, China; ^2^State Key Laboratory for Oxo Synthesis and Selective Oxidation, Lanzhou Institute of Chemical Physics, Chinese Academy of Sciences, Lanzhou, China; ^3^Institute of Microbiology Heilongjiang Academy of Sciences, Harbin, China

**Keywords:** ferulic acid, alkyl ferulic acid esters, structure, antibacterial properties, foodborne pathogens, bacterial membranes

## Abstract

Ferulic acid (FA) is a natural antibacterial agent rich in plants, FA has excellent antioxidant and antibacterial properties. However, because of its short alkane chain and large polarity, FA is difficult to penetrate the soluble lipid bilayer in the biofilm to enter the cell to play an inhibitory role, limiting its biological activity. To improve the antibacterial activity of FA, with the catalytic condition of Novozym 435, four alkyl ferulic acid esters (FCs) with different alkyl chain lengths were obtained by fatty alcohols (including 1-propanol (C3), 1-hexanol (C6), nonanol (C9), and lauryl alcohol (C12)) modification. The effect of FCs on *P. aeruginosa* was determined by Minimum inhibitory concentrations (MIC), minimum bactericidal concentrations (MBC), Growth curves, alkaline phosphatase (AKP) activity, crystal violet method, scanning electron microscopy (SEM), membrane potential, PI, cell contents leakage. Results showed that the antibacterial activity of FCs increased after esterification, and the antibacterial activity significantly increased and then decreased with the extension of the alkyl chain of the FCs. Hexyl ferulate (FC6) showed the best antibacterial activities against *E. coli* and *P. aeruginosa* (MIC for *E. coli* was 0.5 mg/ml, MIC for *P. aeruginosa* was 0.4 mg/ml). And Propyl ferulate (FC3) and FC6 showed the best antibacterial activities S. aureus and *B. subtilis* (MIC for *S. aureus* was 0.4 mg/ml, The MIC of *B. subtilis* was 1.1 mg/ml). In addition, the growth, AKP activity, bacterial biofilm, bacterial cell morphology, membrane potential and cell contents leakage of *P. aeruginosa* after different FCs were investigated, which found that FCs could damage the cell wall of *P. aeruginosa* and showed different effects on the *P. aeruginosa* cell biofilm. FC6 showed the best inhibition on the biofilm formation of *P. aeruginosa* cells, which caused the surface of *P. aeruginosa* cells to be rough and wrinkled. Some *P. aeruginosa* cells showed aggregation and adhesion, even rupture. The membrane hyperpolarization was obvious, which appeared as holes, leading to cell contents leakage (protein and nucleic acid). All these results concluded that the antibacterial activities FCs against foodborne pathogens depended on different fatty alcohol esterification of FA. FC6 showed the best inhibition on *P. aeruginosa* due to its effect on *P. aeruginosa* cell walls and biofilms and the leak of the cell contents. This study provides more practical methods and a theoretical basis for giving full play to the bacteriostatic effect of plant FA.

## 1. Introduction

Infectious diseases caused by bacteria, fungi and viruses have become a major global health problem (Li et al., 2021). Globally, about 4 million people die each year from acute respiratory infections, about 3 million from intestinal infections, about 1.8 from human immunodeficiency virus (HIV), about 1.3 million from tuberculosis, and about 700,000 from malaria (Lozano et al., 2012; Tang et al., 2021). In addition, increasing microbial resistance to conventional drugs has informed studies into new antibacterial agents with broad-spectrum antibacterial activity (Malheiro et al., 2018).

Plant antimicrobials were widely used for their safety, environment-friendly, no drug resistance, and low residue, among other advantages. Studies have revealed that some phenolic substances showed antibacterial activities, for example, they be inserted into the phospholipid layer of bacterial membranes, disrupting the van der Waals interaction between the fatty acyl chains and destroying the phospholipid bilayer and membrane integrity (Burt, 2004; Chauhan et al., 2021; Li et al., 2021). As the ion gradient of the cell membrane is disrupted, important components such as ions and macromolecules are released from within the cell, resulting in the death of the bacteria. FA (4-hydroxy-3-methoxy cinnamic acid, C_10_H_10_O_4_) is a natural antibacterial agent rich in plants such as coffee, rice husk, vanilla bean, wheat bran and rice bran. As a phenolic acid, FA has been proven to have various biological activities that can reduce the risk of serious diseases such as diabetes, cholesterol, heart disease and cancer, and it has excellent antioxidant and antibacterial properties (Burcu et al., 2011; Kot et al., 2018; Pernin et al., 2019). As an antimicrobial agent, FA has a broad antibacterial spectrum, and its antibacterial activities against *pneumococcus*, *Escherichia coli*, *Pseudomonas aeruginosa*, *Shigella Sonneri*, *Bacillus*, *Listeria*, and *Staphylococcus aureus* have been reported (Saharkhiz et al., 2012; Barbara et al., 2015; Shi et al., 2016, 2018, 2019).

**Figure 1:**
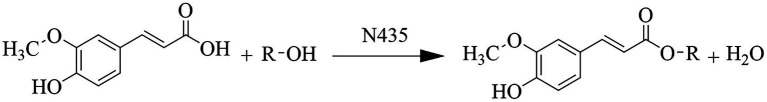
Ferulic acid reacts with fatty alcohol to form ferulic ester equation.

However, because of its short alkane chain and large polarity, FA is difficult to penetrate the soluble lipid bilayer in the biofilm to enter the cell to play an inhibitory role, limiting its biological activity (Stamatis et al., 2001). It was found that FA esterified by fatty alcohols (Figure 1) can improve the lipid solubility of the molecule and the antibacterial activity of FA, but there is a lack of detailed comparison of ferulic acid and ferulic acid with different alkane chain lengths on the antibacterial activities and the effects on the bacterial cell wall and cell membrane. Bacteria were divided into Gram-positive and Gram-negative bacteria based on Gram staining, with the main difference between the two being the structure of the cell wall, so 2 g-positive and 2 g-negative food-borne pathogens were selected as test bacteria in this experiment. In this study, four FCs with different alkyl chain lengths were prepared by fatty alcohols (C3, C6, C9 and C12) modification, and the effect of FCs molecules with different alkane chains on antibacterial activities against foodborne pathogens (*E. coli*, *P. aeruginosa*, *S. aureus*, and *B. subtilis*) and their mode of action were investigated.

## 2. Materials and methods

### 2.1. Materials

Ferulic acid, 1-propanol (C3), 1-hexanol (C6), nonanol (C9), and lauryl alcohol (C12) with >98% purity were purchased from Aladdin Biochemical Technology Co., LTD. (Shanghai, China). Novozym 435 (A Candida antarctica lipase B immobilized on acrylic resin) was purchased from Novozymes Biotechnology LTD. (Denmark). 4 Å molecular sieve and n-hexane (moisture ≤0.02%) were purchased from Guangfu Fine Chemical Research Institute (Tianjin, China). The alkaline phosphatase (AKP) kit and BCA protein quantitative kit were purchased from Dalian Meilun Biotechnology Co., LTD. (Dalian, China). P-Iodonitrotetrazolium Violet (INT) was purchased from Macklin Biochemical Co., LTD. (Shanghai, China). 2.5% glutaraldehyde was purchased from Leagene Biotechnology Co., LTD. (Beijing, China). Rhodamine 123 was purchased from Biotopped Life sciences. (Beijing, China). Propidium iodide (PI) was purchased from Saiguo Biotechnology Co., LTD. (Guangzhou, China). Ferulic acid, 1-propanol, 1-hexanol, nonanol, lauryl alcohol, Novozym435 and other organic solvents were treated with a 4 Å molecular sieve for at least 24 h before use.

### 2.2. Bacterial strains and culture conditions

*Escherichia coli* ATCC 25922, *Pseudomonas aeruginosa* ATCC 27853, *Staphylococcus aureus* ATCC 25923 and *Bacillus subtilis* were gift samples from the Institute of Microbiology Heilongjiang Academy of Sciences. The strains preserved in the tube were removed from the −80°C refrigerator and thawed overnight in a 4°C refrigerator. All strains were activated by Tryptone Soy broth (TSB) medium (Hangzhou Microbe 164 Reagent Co., LTD., China) at 37°C in an orbital air shaking bath (180 rpm) for 24 h and maintained on TSA slants at 4°C.

### 2.3. Preparation of ferulic acid alkyl esters

In this experiment, four fatty alcohols (C3, C6, C9, C12) were selected for esterification with FA to obtain four kinds of FCs with different alkane group lengths. The method described by Shi et al. (2017) was modified slightly. Briefly, FA and fatty alcohol (molar ratio, 1:8) was dissolved in 10 ml n-hexane solvent in a 50 ml triangular bottle, 80 mg of Novozym435 lipase and 10 molecular sieves (4 Å) were added. The reaction was carried out in an orbital air shaking bath (150 rpm) at 50°C for 7 days. The n-hexane solvent was then removed under reduced pressure, and the crude residue was purified by column chromatography on a column (1.5 × 20 cm) packed with silica gel 60 (230–400 mesh, Merck) using hexane: ethyl acetate 8:2 (v/v) as eluent.

### 2.4. NMR analysis and mass spectrometry

The molecular structure of the FCs was determined by NMR (model AVANCEII400, Swiss-Brock). FCs were weighted with 4 mg for ^1^H-NMR determination and 15 mg for ^13^C-NMR determination. After the FCs were dissolved in 0.6 ml deuterium chloroform (CDCl_13_), the solution was quickly transferred to a clean and dry nuclear magnetic tube (5 mm, 7 inches) for determination. The propyl ferulate (FC3) hexyl ferulate (FC6)nonyl ferulate (FC9) Lauryl ferulate (FC12) of ^1^H NMR and ^13^C NMR were all 400 MHz and 100 MHz, respectively (Shi et al., 2019).

Fourier Transform high-resolution mass spectrometry (Velos, ThermoFisher, United States) was used to analyze FCs molecular weight. Five hundred micro liter of methanol was added to a 1.5 mL centrifuge tube, and a trace of the tested sample was dipped into a capillary tube and then dissolved in chromatograde methanol. Methanol solution containing FCs was extracted with an injection needle and injected into the pump for determination. The ESI-MS(m/z) of FC3, FC6, FC9 and FC12 were 237.1[M + H] + ，279.2[M + H]+, 321.2 [M + H]+, 363.3[M + H]+, respectively. The NMR results of FC3, FC6, FC9 and FC12 were shown in Table 1.

**Table 1:** Minimum inhibitory concentration MIC (mg/ml) and minimum bactericidal concentration MBC (mg/ml) values of FCs against different pathogens bacterial strains.

Name of compound	*R*	*c* Log *P**^a^*		*Escherichia coli* (G^−^)	*Pseudomonas aerugino* (G^−^)	*Staphylococcus aureus* (G^+^)	*Bacillus subtilis* (*G+*)
FA	H	1.4212	MIC	1.2	1.0	0.7	1.4
			MBC	2.5	3.0	1.5	3.5
FC3	(CH_2_)_2_CH_3_	2.7052	MIC	0.8	0.7	0.4	1.1
			MBC	3.0	3.0	2.0	4.0
FC6	(CH_2_)_5_CH_3_	4.2922	MIC	0.5	0.4	0.4	1.1
			MBC	3.0	3.0	2.0	4.0
FC9	(CH_2_)_8_CH_3_	5.8792	MIC	1.0	0.9	0.8	1.3
			MBC	3.5	4.0	2.5	4.5
FC12	(CH_2_)_11_CH_3_	7.4662	MIC	3.3	2.8	2.6	3.5
			MBC	5.0	4.5	4.0	5.0

a ChemDraw 20.0 software was used for theoretical estimation.

FC3: 1H NMR (CDCl3, 400 MHz) δ 7.61 (d, J = 16.0 Hz, 1H), 7.07–7.01 (m, 2H), 6.29 (d, J = 16.0 Hz, 1H), 6.03 (s, 1H), 4.15 (t, J = 8.0 Hz, 1H), 3.90 (s, 1H), 1.76–1.67 (m, 2H), 0.98 (t, J = 8.0 Hz, 3H). 13C NMR (CDCl3, 100 MHz) δ 167.56, 148.09, 148.07, 146.92, 144.81, 126.89, 122.97, 115.39, 114.87, 109.46, 66.05, 55.85, 22.08, 10.44.

FC6: 1H NMR (400 MHz, CDCl3) δ 7.61 (d, J = 16.0 Hz, CH), 7.08–7.03 (m, 2CH), 6.91 (d, J = 7.6 Hz, CH), 6.29 (d, J = 16.0 Hz, CH), 5.88 (s, OH), 4.19 (t, J = 5.6 Hz, CH2), 3.93 (s, CH3), 1.69–1.33 (m, 4CH2), 0.90 (s, CH3). 13C NMR (100 MHz, CDCl3) δ 167.6, 148.0, 146.9, 144.8, 127.2, 123.2, 115.8, 114.8, 109.4, 64.8, 56.1, 31.6, 28.9, 25.8, 22.7, 14.2.

FC9: 1H NMR (400 MHz, CDCl3) δ 7.60 (d, J = 16.0 Hz, CH), 7.08–7.03 (m, 2CH), 6.91 (d, J = 8.4 Hz, CH), 6.29 (d, J = 15.6 Hz, CH), 5.95 (t, OH), 4.18 (t, 6.4 HZ, CH2), 3.92 (s, CH3),1.73–1.27(m, 7CH2). 0.88 (t, 6.8 HZ,CH3). 13C NMR (100 MHz, CDCl3) δ 167.4, 147.9, 146.7, 144.6, 127.0, 123.0, 115.7，114.7, 109.3, 64.6, 55.9, 31.9, 29.5, 29.3, 29.2, 28.8, 26.0, 22.7,14.1.

FC12: 1H NMR (CDCl3, 400 MHz) δ 7.61 (d, J = 16.0 Hz, 1H), 7.08–7.03 (m, 2H), 6.91 (d, J = 8.0 Hz, 1H), 6.29 (d, J = 16.0 Hz, 1H), 5.89 (s, 1H), 4.19 (t, J = 4.0 Hz, 2H), 3.92 (s, 1H), 1.73–1.66 (m, 2H), 1.26 (s, 18H), 0.88 (t, J = 4.0 Hz, 3H). 13C NMR (CDCl3, 100 MHz) δ 167.59, 148.08, 146.93, 144.82, 127.06, 123.11, 115.61, 114.88, 109.45, 64.74, 55.97, 32.00, 29.74, 29.72, 29.68, 29.63, 29.44, 29.39, 28.84, 26.08, 22.77, 14.20.

### 2.5. Minimum inhibitory concentrations and minimum bactericidal concentrations determination

The method described by Aljawish et al. (2016) was slightly modified in this step. MIC and MBC were determined by the broth microdilution method combined with the INT indicator method. The FCs solution was prepared with anhydrous ethanol. Single colonies of *P. aeruginosa* were inoculated in TSB medium and cultured at 37°C for 14–16 h. The culture solution was centrifuged(4,000 × g, 4°C, 6 min), and the collected bacteria were washed with sterile 0.01 mol/l PBS (pH 7.4). The cultures obtained were diluted with TSB to obtain a 0.5 MCF (1 × 10^8^ CFU/ml) and diluted 10 times.

Ninety six-well microplates were filled with 160 μL TSB medium, and then 20 μL of diluted bacterial strains and 20 μL of each FCs concentration were added to the wells. Two controls were used in two columns that contained 160 μL TBS medium and 20 μL of diluted bacterial strains: one with 20 μl of sterile distilled water and another with 20 μl of Anhydrous ethanol. Tests were carried out in triplicate. After being cultured at 37°C for 14–16 h, 10 μL 0.4% (w/v) INT was added to the wells as an indicator and cultured at 37°C for 30 min in the dark. The colorless tetrazolium salt acts as an electron acceptor and is reduced to a red-colored formazan product by biologically active organisms. When bacterial growth was inhibited, the solution in the well remained colorless after incubation with INT. When bacterial growth was inhibited (no evidence of growth), MIC values were recorded as the minimal concentration of FCs concentration. For MBC determination, 50 μl from each well without INT was spread on TSA agar plates and incubated at 37°C for 24–48 h. At least two duplicate tests were performed with each strain and antibacterial compound.

### 2.6. Growth curves

The method described by Campion et al. (2017) has been slightly modified. Single colonies of *P. aeruginosa* were inoculated in TSB medium and cultured at 37°C for 14–16 h in an orbital air shaking bath (180 rpm). The culture solution was centrifuged(4,000 × g, 4°C, 6 min), and the collected bacteria were washed with sterile 0.01 mol/l PBS (pH 7.4). The cultures obtained were diluted with TSB to obtain a 0.5 MCF (1 × 10^8^ CFU/ml) and diluted 100 times. FA, FC3, FC6, FC9 and FC12 were added, respectively, until the final concentration was 0.4 mg/ml, and no drug was added as control. The absorbance values were measured every 2 h at 600 nm after 24 h culture at 37°C. The growth curve was plotted with the culture time as the abscissa and the OD_600_ value as the ordinate.

### 2.7. Determination of cell wall integrity

The method described by Shu et al. (2019) was slightly modified for this procedure. FA, FC3, FC6, FC9 and FC12 were, respectively, added into the bacterial suspension (As described in 2.6) to reach the final concentration of 0.4 mg/ml, with no drug added as the control. Under the condition of 37°C and 160 rpm at 0, 1, 2, 4, 6, and 8 h, the culture solution was centrifuged(4,000 × g, 4°C, 6 min)，and take the supernatant to detect the AKP activity.

### 2.8. Determination of bacterial biofilm by crystal violet method

The method described by Djordjevic et al. (2002) has been slightly modified. FA, FC3, FC6, FC9 and FC12 were, respectively, added into the bacterial suspension (As described in 2.6) to reach the final concentration of 0.4 mg/ml, with no drug added as the control. Two hundred microliter was added to the 96-well plate, incubated at 37°C for 48 h, and the single bacterial cells were removed. Each well was gently cleaned with sterile 0.01 mol/l PBS (pH7.4) thrice, and added 200 μL formaldehyde solution to each well to fix for 5 min after drying. Then, 200 μL 0.1%crystal violet solution was added to each well, incubated at room temperature for 10 min, washed with sterile 0.01 mol/LPBS (pH 7.4) thrice to remove excess crystal violet, and dried at room temperature. Finally, 200 μL 95% ethanol was absorbed and added to each well, and the OD value of 600 nm was read. Use the following formula to calculate the percentage inhibition of bacterial biofilm formation.


$$\mathrm{Inhibition rate}\;\left(\%\right)=\left[1-\frac{E_{i}}{E_{0}}\right]\times 100\%$$

where E_i_ and E_0_ are absorbance values treated with or without FCs, respectively.

### 2.9. Observation of microstructures by scanning electron microscopy

The method described by Shao et al. (2018) has been slightly modified. FA, FC3, FC6, FC9, and FC12 were, respectively, added into the bacterial suspension (As described in 2.6) to reach the final concentration of 0.4 mg/mL, with no drug added as the control. Under the condition of 37°C and 160 rpm at 2 h, the culture solution was centrifuged(4,000 × g, 4°C, 6 min) to take the bacteria. 2.5% glutaraldehyde was used to fix bacteria for more than 1.5 h under a 4°C refrigerator. The collected bacteria were flushed with 0.1 mol/l PBS (pH 7.2) twice (10 min each time), dehydrated with 50, 70, and 90% ethanol once for 10 min, and dehydrated with 100% ethanol twice for 10 min. After dehydration, the bacterial cells were vacuum freeze-dried and coated with gold spray. A scanning electron microscope (model SU8010, Hitachi, Japan) was used to observe the morphological changes of bacteria cells.

### 2.10. Measurement of membrane potential

This step was performed by slightly modifying the method of Jung et al. (2015). FA, FC3, FC6, FC9, and FC12 were, respectively, added into the bacterial suspension (As described in 2.6) to reach the final concentration of 0.4 mg/mL, with no drug added as the control. Under the condition of 37°C and 160 rpm at 3 h, the bacteria were collected, washed and dissolved with PBS. The Rhodamine 123 (2 μg/mL) was added, and the samples were washed three times and then suspended in PBS after 30 min in the dark. The average fluorescence intensity of the cell suspension was measured with a multi-functional enzyme spectrometer at the excitation wavelength of 489 nm, the emission wavelength of 530 nm and the slit width of 10 nm.

### 2.11. Evaluation of membrane permeability

#### 2.11.1. Fluorescent detection of PI

The method described by Park and Kang (2013) has been slightly modified. FA, FC3, FC6, FC9 and FC12 were, respectively, added into the bacterial suspension (As described in 2.6) to reach the final concentration of 0.4 mg/ml, with no drug added as the control. Under the condition of 37°C and 160 rpm at 3 h, 0.3 mL PI(1 mg/ml)solution was added to the experimental group and control group, respectively, and incubated at 37°C for 10 min. The bacteria liquid’s fluorescence values (excitation light 485 nm, emission light 500–800 nm, slit width 5 nm) with the above concentration gradient were measured successively, and the data were read.

#### 2.11.2. Determination of intracellular protein leakage

FA, FC3, FC6, FC9, and FC12 were, respectively, added into the bacterial suspension (As described in 2.6) to reach the final concentration of 0.4 mg/ml, with no drug added as the control. The supernatant was cultured at 37°C and 160 rpm for 2, 4, 6, and 8 h, filtered by 0.22 μm microporous membrane. The protein content was determined by BCA protein quantitative kit.

#### 2.11.3. Determination of cell contents leakage

The method described by Zhao et al. (2015) was slightly modified. FA, FC3, FC6, FC9, and FC12 were, respectively, added into the bacterial suspension (As described in 2.6) to reach the final concentration of 0.4 mg/mL, with no drug added as the control. Cultured at 37°C and 160 rpm for 4 h, the supernatant was taken, and the value of 260 nm was determined by ultraviolet spectrophotometer.

### 2.12. Statistical analysis

All experiments were performed in triplicate. Data were presented as mean ± SD. Data were analyzed for analysis of variance (ANOVA) by SPSS software to determine the least significant differences (*p* < 0.05).

## 3. Results and discussion

### 3.1. MIC and MBC evaluation

MIC is the lowest inhibitory concentration of the drug that can inhibit the growth of pathogenic bacteria in the culture medium after 14–18 h of bacterial culture *in vitro*. On the other hand, the MBC was defined as the minimal concentration of antibacterial compound that results in 99.9% dead cells in the primary inoculum (no colony was observed on TSA agar plates) (Naraysanan et al., 2021). In this experiment, the MIC was determined by broth dilution and INT rapid detection method. Aljawish et al. (2016) confirmed that the INT rapid detection results were consistent with the traditional optical density detection results. As shown in Table 1, FA and FCs showed antibacterial activities against four Gram-negative and Gram-positive pathogens. FC6 showed the best antibacterial activity against *E. coli* and *P. aerugino*, with MIC values of 0.5 mg/ml s and 0.4 mg/ml, MBC value of both 3.0 mg/ml. In addition, FC3 and FC6 showed the best antibacterial activity against *S. aureus* and *B. subtilis*, with MIC values of 0.4 mg/ml and 1.1 mg/ml, MBC values of 2.0 mg/ml and 4.0 mg/ml. Borges et al. (2012) used ferulic acid as a bacteriostatic agent to study its bacteriostatic activity on *P. aeruginosa*, *E. coli*, *Listeria*, and *S. aureus* and also confirmed that ferulic acid had an inhibitory effect on *P. aeruginosa*, *E. coli*, and *S. aureus*. In addition, Pinheiro et al. (2021) used ferulic acid and its four esterified derivatives (methyl, ethyl, propyl and butyl) for antibacterial activity against *S. aureus*. These researches confirm our results that FA can have antibacterial functionalities in using FA as a potential preservative in the food industry. In the 4 pathogenic bacteria strains, the determination of antibacterial activity shows that after esterification of FA, bacteriostatic activity increased, but with the extension of antibacterial compounds alkyl chain, the bacteriostatic activity increased first and then, for *E. coli* and *P. aeruginosa*, FC6 bacteriostatic activity of the best, for *S. aureus* and *B. subtilis*, best FC3 and FC6 antibacterial activity.

### 3.2. Influences on the growth curve of *Pseudomonas aeruginosa*

**Figure 2:**
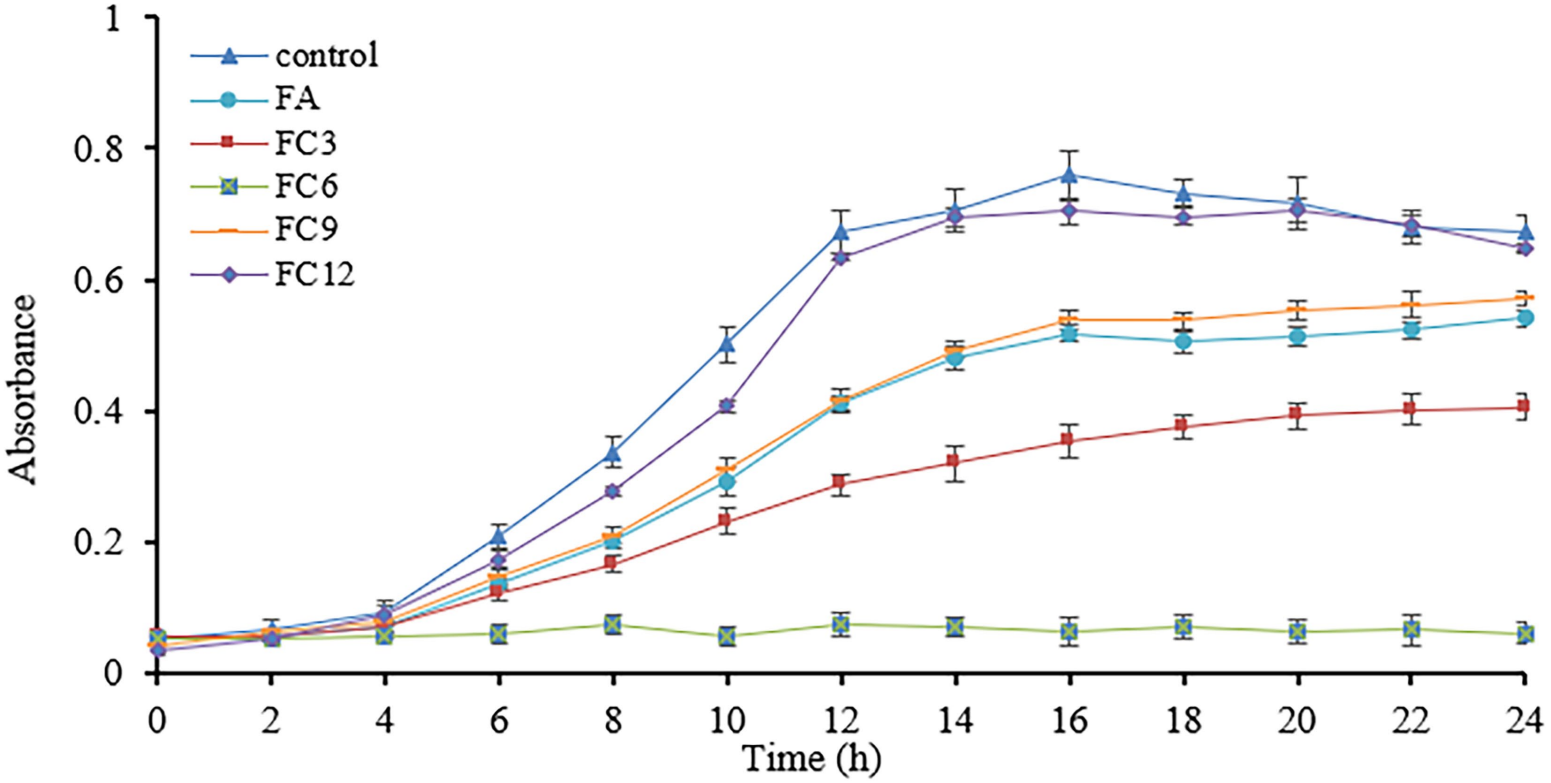
Effect on the growth curve of *Pseudomonas aeruginosa.*

Using *P. aerugino* as the indicator pathogen, the antibacterial activity of 0.4 mg/ml FA, FC3, FC6, FC9 and FC12, respectively, on the growth were investigated, and the results are shown in Figure 2 *P. aeruginosa* of the control group entered the logarithmic growth stage after culture for 4 h, and showed an increasing trend to the stable stage until 16 h. In the concentration of 0.4 mg/ml FC6 (1 × MIC) treatment group, the growth of the *P. aeruginosa* was completely suppressed—the growth of *P. aeruginosa* treated with FC3 was inhibited, and the logarithmic growth period was prolonged. However, FC9 and FC12 showed insignificant growth inhibition on *P. aeruginosa*. The effect of FCs on the growth curve of *P. aeruginosa* further verified the antibacterial activity of FCs against *P. aeruginosa*. Merkl (2010) reported that phenolic acid fatty alcohol esters could show a broad spectrum of antibacterial activity. In particular, *P. aeruginosa* (G-) and *S. aureus* (G+) were more sensitive to FC6, with MIC values of 0.4 mg/ml. Shi et al. (2017) took ferulic acid and fatty alcohol of ferulic acid from C4 to C12 as bacteriostatic agents to study the bacteriostatic activity against *E. coli* and *L. monocytogenes*, and the results showed that C6 had the best bacteriostatic activity, which was consistent with the results of this study. The possible explanations are as follows: the antibacterial activity of FCs is closely related to the species of bacteria and the length of the carbon chain of FCs (Kubo et al., 2004). We also found that, *E. coli*(G-) (MIC = 0.5 mg/mL), *P. aerugino*(G-) (G-) (MIC = 0.4 mg/ml), *S. aureus*(G+)(MIC = 0.4 mg/mL) was more sensitive to FC6 than *B. subtilis*(G+)(MIC = 1.1 mg/mL). Borges et al. (2012) confirmed that ferulic acid had more effective antibacterial activity against Gram-negative bacteria (*E. coli*, *P. aerugino*) than Gram-positive bacteria.

### 3.3. Effect on the integrity of *Pseudomonas aeruginosa* cell wall

**Figure 3:**
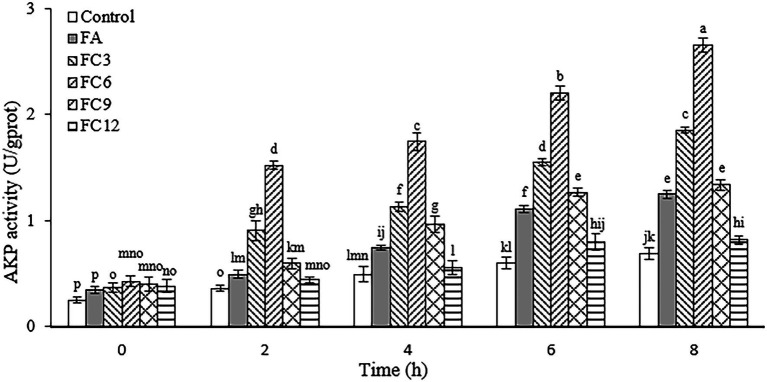
Effect on the integrity of *Pseudomonas aeruginosa* cell wall.

Cell walls can effectively prevent foreign substances, such as antibiotics and bacteriostatic agents, from entering cells. AKP exists between the cell membrane and the cell wall and plays an important role in the metabolism of substances in organisms. Therefore, the integrity of the cell wall can be reflected by detecting AKP outside the cell (Shu et al., 2019). Figure 3 shows the AKP leakage of *P. aerugino* after being treated by antibacterial agents FA, FC3, FC6, FC9 and FC12. After FA and different FCs were treated with the same inhibitory concentration of *P. aerugino*, AKP activity increased with the extension of culture time. It should be noted that FC6-treated *P. aerugino* rapidly increased AKP activity within 2 h and showed the largest AKP activity at 8 h, with an activity of 2.66 U/gprot. Based on these results, it was speculated that FCs increased the permeability of the cell wall and damaged the integrity of the cell wall, thus allowing AKP to leak into the cell culture medium. This observation agrees with a recent review by Aldulaimi (2017) that FA acts synergistically with other phenolics like gallic acid against notable pathogens like *P. aerugino* cell wall, resulting in content leakage. Bearing in mind that the threats posed by this and other foodborne pathogens are still major threats to public health, it will be interesting to investigate synergistic antibacterial effects in the future.

### 3.4. Effects on *Pseudomonas aeruginosa* biofilm formation

**Figure 4:**
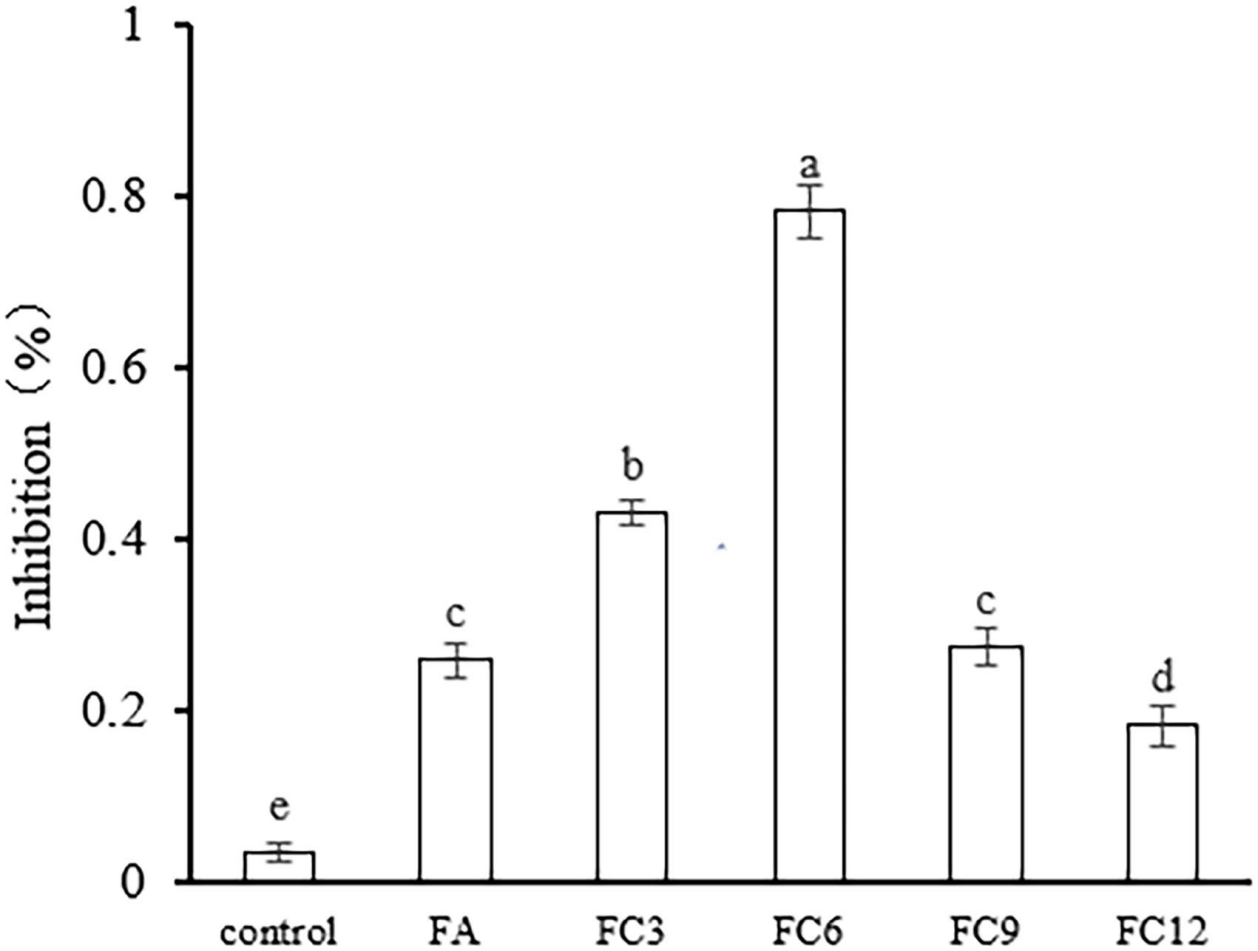
Effect on *Pseudomonas aeruginosa* biofilm formation.

The inhibition of FA, FC3, FC6, FC9, and FC12 on FA biofilm formation was evaluated by crystal violet staining. The results are shown in Figure 4. According to the inhibitory effect on biofilm formation, the order is FC6 (78%) > FC3 (43%) > FC9 (27%) > FA (26%) > FC12 (18%). The results indicated that FC6 had the strongest inhibition effect on forming *P. aerugino* biofilm at the same concentration. Floating motion plays an important role in the pathogenic ability of *P. aerugino*, which can initiate the adhesion of bacteria to the adhesive surface and contribute to the formation of *P. aerugino* biofilm. Therefore, these partial results suggest that the inhibition of this motion may be one of the reasons for the reduction of biofilm formation(Diao et al., 2018; Zhao et al., 2019). Furthermore, a recent comparative study by Xu et al. (2022) revealed that FA had a greater biofilm repression effect than *p-*coumaric acid (*p-*CA) against pathogenic *Salmonella enteritidis* by binding to proteins involved in flagella motility. In addition to maintaining high antibiofilm effectiveness in simulated conditions, both phenolics were forwarded as candidates for modifying this notable pathogen’s phenotypic and molecular structures. This report supports our observation that further studies will prove valuable in enhancing the pathogen-inhibiting applications of FA.

### 3.5. Influence on somatic morphology of *Pseudomonas aeruginosa* bacteria

**Figure 5:**
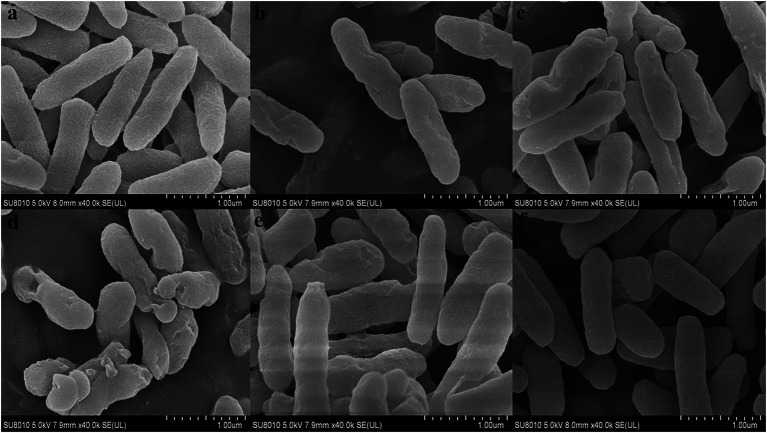
Effect on morphology of *Pseudomonas aeruginosa* cells. **(A)** Shows the Control group. **(B)** Shows the experimental group treated with FA. **(C)** Shows the experimental group treated with FC3. **(D)** Shows the experimental group treated with FC6. **(E)** Shows the experimental group treated with FC9. **(F)** Shows the experimental group treated with FC12.

In order to further observe the effects of FA, FC3, FC6, FC9 and FC12 on the integrity of *P. aerugino* cell wall and membrane, the morphological changes of *P. aerugino* cells treated with an antibacterial agent for 2 h were observed by scanning electron microscopy. As shown in Figure 5, the *P. aerugino* cells in the control group (Figure 5A) were complete, full in shape, clear in outline, rod-shaped, and relatively smooth in surface, showing a typical Gram-negative bacterial morphological structure. In the *P. aerugino* cells treated by FA, FC3 and FC9 (Figures 5B, C, E), thalli were deformed, their surfaces collapsed and crumpled, and their surfaces were rough. The surface of *P. aerugino* treated by FC12 (Figure 5F) showed no change compared with the control group, only some cells were shrunken. The FC6-treated cells (Figure 5D) showed rough surface, severe shrinkage, aggregation, and adhesion; some cells even ruptured. Dasagrandhi et al. (2018) treated *P. aerugino* cells with ferulic acid-grafted chitosan (CFA) with 1 × MIC concentration to deform the bacterial cells and gather them together in large numbers, causing damage to the bacterial membrane. This agrees with the current study’s findings, and thus future experiments may need to investigate if there is a molecular basis for these processes, as previously posited by Xu et al. (2022).

### 3.6. Influence on FA membrane potential

**Figure 6:**
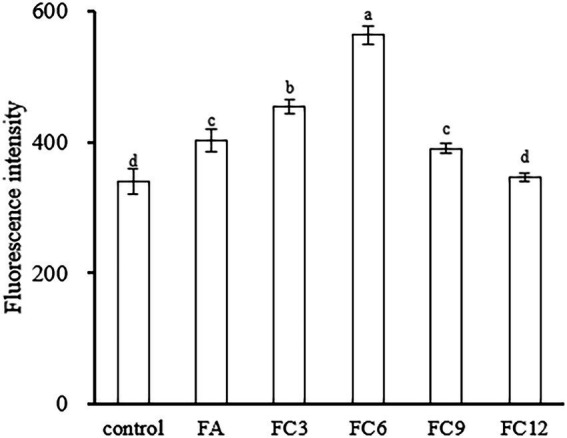
Effect on membrane potential of *Pseudomonas aeruginosa.*

Membrane potential is the potential difference between the inside and outside of a biological cell membrane, and maintaining normal membrane potential is of great significance for ATP production and cell function maintenance (Xu et al., 2016). Rhodamine 123 is a lipophilic cationic dye that can enter the cell matrix by transmembrane potential, the size of which can be expressed by its fluorescence intensity. Under normal circumstances, the voltage inside the membrane is usually lower than outside the membrane. If the membrane potential decreases, it means that the membrane is depolarized; otherwise, if it increases, it means that hyperpolarization occurs (Kiran and Yarlagadda, 2020. Figure 6 shows the effects of FA and FCs on the membrane potential of *P. aerugino*. As shown in Figure 6, both FA and FCs can hyperpolarize the membrane of *P. aerugino*, and FC3 and FC6 have significant effects on the membrane potential of *P. aerugino*. Control group’s fluorescence intensity of 340.33 ± 18.77 on average, after FC3 and FC6 treatment, and the fluorescence intensity increased to 453.67 ± 10.41 and 564.33 ± 14.01 respectively, compared with the control group, with a growth of 33 and 66% respectively, compared with the control group, both were extremely significant differences. The results showed that FC6 had more serious effects on *P. aeruginosa*. Shang et al. (2017) showed that the target of the antibiotic drug chensinin-1b is the plasma membrane through the cell intimal permeability experiment results. Chensinin-1b can increase the membrane potential of MRPA0108, increasing membrane permeability, leakage of contents, and the death of bacteria.

### 3.7. Evaluation of membrane permeability of *Pseudomonas aeruginosa*

#### 3.7.1. Propidium iodide uptake test

**Figure 7:**
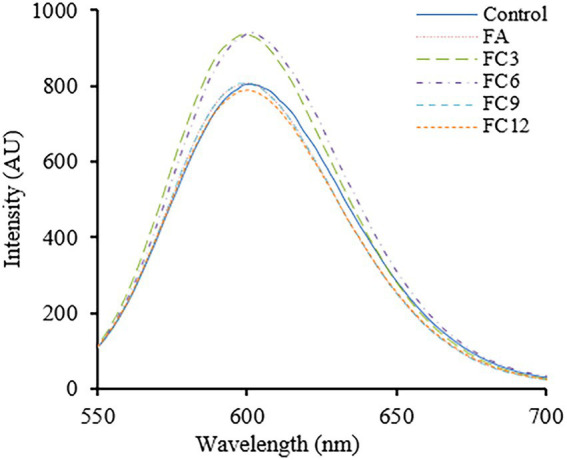
Effect on membrane integrity of *Pseudomonas aeruginosa.*

PI can be embedded into DNA double-stranded molecules, making them fluoresce, a fluorescent probe. Under normal physiological conditions, PI cannot enter cells, but when the cell membrane breaks, PI can cross the cell membrane and enter the cell, binding to DNA. Therefore, the integrity of the cell membrane can be judged by measuring the fluorescence intensity after PI interacts with DNA (Chakotiya et al., 2017). Figure 7 shows the effects of FA and FCs on cell membrane integrity. As shown in Figure 7, in the range of wavelength 550–700 nm, the fluorescence intensity of FA 4 h under the action of FC3 and FC6 was significantly stronger than that of the control group, indicating that FC3 and FC6 could be effective against *P. aerugino* membrane by damaging its integrity and preventing further disease spread. These observations were similar to earlier findings that FA and other phenolics have growing bactericidal effects against foodborne pathogens (Diao et al., 2018; Xu et al., 2022). Another recent research by Kong et al. (2023) revealed that the FA ester derivatives had substantial *in vitro* and *in vivo* inhibitory effects against *Alternaria alternata,* a major fungal pathogen causing black spot disease of Korla pear has resulted in exponential losses. Thus, further *in vivo* mechanistic studies must be conducted to show more potential for FA and its ester derivatives in the food, agricultural, and biomedical sectors.

#### 3.7.2. Impact on leakage of *Pseudomonas aeruginosa* contents

**Figure 8:**
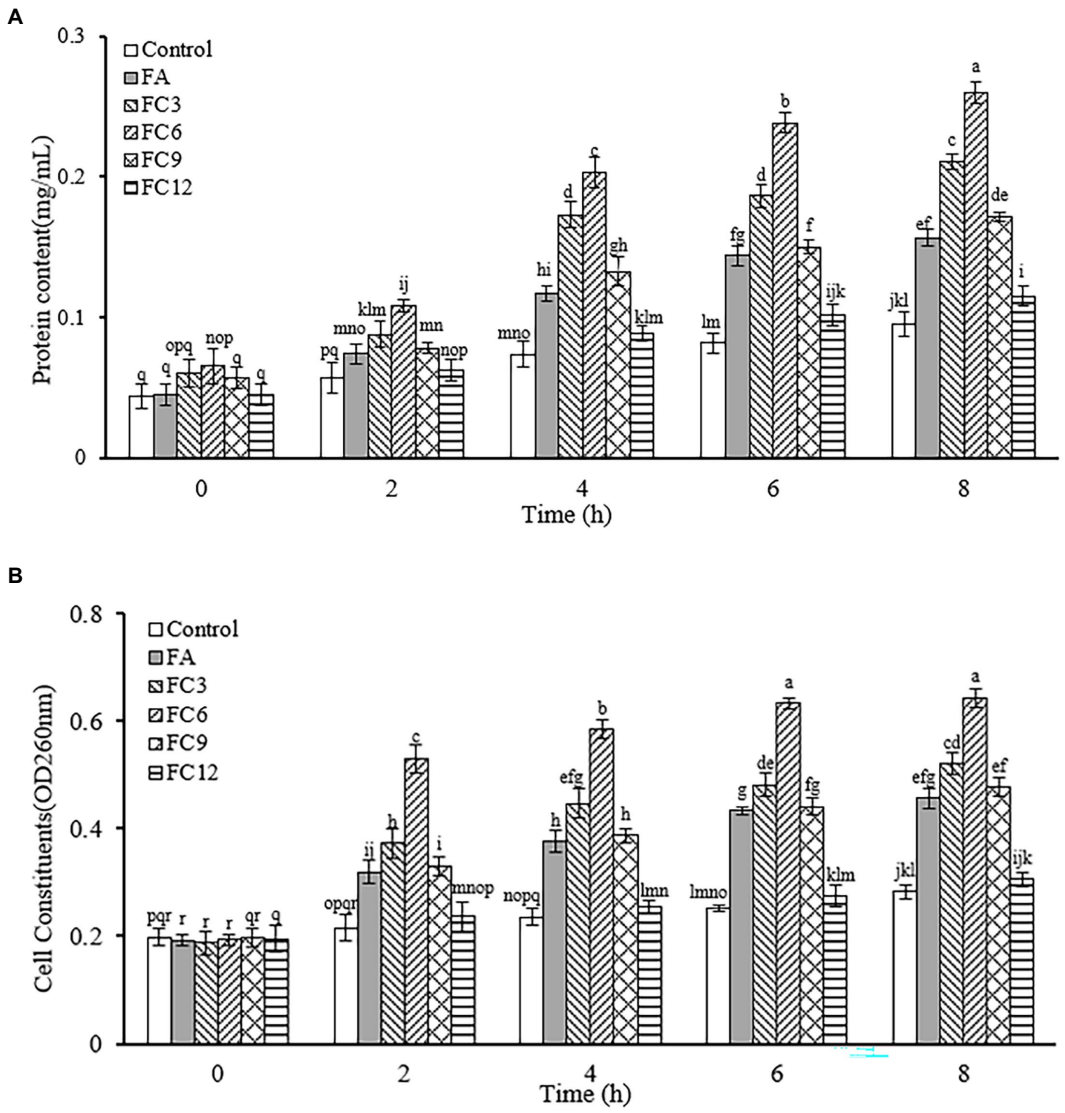
Effect on leakage of *Pseudomonas aeruginosa* cell contents. **(A)** Effect on protein leakage in *Pseudomonas aeruginosa* cells. **(B)** Effect on nucleic acid leakage of *Pseudomonas aeruginosa* cells.

Nucleic acid, protein, Na^+^, K^+^ and other cell contents play an important role in maintaining the normal physiological function of cells. In normal cells, cell contents will not cross the cell membrane. When the bacterial cell membrane is destroyed, some macromolecules will enter the external environment through the cell membrane, resulting in the increase of the absorbance of the external environment at 260 and 280 nm wavelengths. Thus, this wavelength range can be used to measure cell content leakage. To determine the permeability of the cell membrane. Figure 8 shows the influence of FA, FC3, FC6, FC9 and FC12 on the leakage of *P. aerugino* contents at different times. Figure 8A shows the leakage of protein, and Figure 8B shows the leakage of nucleic acid. As shown in Figure 8A, the *P. aerugino* treated by FA, FC3, FC6, FC9 and FC12 all have different levels of protein leakage, and the *P. aerugino* protein leakage treated by FC6 is the largest. With the prolongation of the antibacterial agent and *P. aerugino*, the protein content in bacterial suspension increased gradually. At 4 h, the protein content after FC6 action on *P. aerugino* was 0.203 mg/ml, 2.94 times that of the control group (0.069 mg/ml). As shown in Figure 8B, at 4 h, after FC6 acted on *P. aerugino*, the leakage of nucleic acid was significantly higher than that of the control group and other FCs, and the absorbance was 2.48 times that of the control group. Combined with Figures 8A,B, it can be seen that FCs can cause the leakage of macromolecular nucleic acids and proteins in *P. aerugino* cells to varying degrees, indicating that FCs change the permeability and integrity of cell membrane, which is consistent with the above results of cell integrity. Li et al. (2020) reported that the integrity of the cell membrane would be damaged after the antibacterial peptide CM4 was applied to *P. aerugino* cells, resulting in nucleic acid and protein leakage.

## 4. Conclusion

Antimicrobial resistance posed by foodborne pathogens makes them a constant threat to global public safety, prompting studies into novel ways of overcoming this challenge. In this study, MIC and MBC values of the 4 pathogenic bacteria(*E. coli, P. aerugino, S. aureus*, *and B. subtilis)* showed that after esterification, the antibacterial activity increased, but with the extension of the alkyl chain of antibacterial compounds, the antibacterial activity increased first and then decreased. FCs can destroy the cell wall of Pseudomonas aeruginosa and affect the biofilm of *P. aerugino* cells to varying degrees. Among them, FC6 inhibits the formation of the biofilm of *P. aerugino* cells the most, making the surface of *P. aerugino* cells rough and wrinkled, and showing aggregation and adhesion. Some cells even rupture. The integrity of the cell membrane is damaged, and the contents of the cell (protein, nucleic acid) are leaked. The effect of FCs on the biofilm of *P. aerugino* cells further indicated that the antibacterial activity of FA increased after esterification, but the antibacterial activity increased first and then decreased with the extension of the alkyl chain of antibacterial compounds. However, the application of FCs in the food system needs further research.

## Data availability statement

The original contributions presented in the study are included in the article/Supplementary material, further inquiries can be directed to the corresponding author.

## Author contributions

WS and JX conceived and designed the experiments. WS and CY performed the experiments. JX and CX supervised the project. WS analyzed the data. WS, JX, CY, and YP wrote the paper. All authors contributed to the article and approved the submitted version.

## Funding

This study was supported by the Natural Science Foundation of Heilongjiang Province (LH2020C063).

## Conflict of interest

The authors declare that the research was conducted in the absence of any commercial or financial relationships that could be construed as a potential conflict of interest.

## Publisher’s note

All claims expressed in this article are solely those of the authors and do not necessarily represent those of their affiliated organizations, or those of the publisher, the editors and the reviewers. Any product that may be evaluated in this article, or claim that may be made by its manufacturer, is not guaranteed or endorsed by the publisher.
